# Criticality as a Set-Point for Adaptive Behavior in Neuromorphic Hardware

**DOI:** 10.3389/fnins.2015.00449

**Published:** 2015-12-01

**Authors:** Narayan Srinivasa, Nigel D. Stepp, Jose Cruz-Albrecht

**Affiliations:** ^1^Information and System Sciences Lab, Center for Neural and Emergent Systems, HRL Laboratories LLCMalibu, CA, USA; ^2^Microelectronics Laboratory, HRL Laboratories LLCMalibu, CA, USA

**Keywords:** neuromorphic electronics, adaptive behavior, criticality, spiking, self-organization, synaptic plasticity, homeostasis, current balance

## Abstract

Neuromorphic hardware are designed by drawing inspiration from biology to overcome limitations of current computer architectures while forging the development of a new class of autonomous systems that can exhibit adaptive behaviors. Several designs in the recent past are capable of emulating large scale networks but avoid complexity in network dynamics by minimizing the number of dynamic variables that are supported and tunable in hardware. We believe that this is due to the lack of a clear understanding of how to design self-tuning complex systems. It has been widely demonstrated that criticality appears to be the default state of the brain and manifests in the form of spontaneous scale-invariant cascades of neural activity. Experiment, theory and recent models have shown that neuronal networks at criticality demonstrate optimal information transfer, learning and information processing capabilities that affect behavior. In this perspective article, we argue that understanding how large scale neuromorphic electronics can be designed to enable emergent adaptive behavior will require an understanding of how networks emulated by such hardware can self-tune local parameters to maintain criticality as a set-point. We believe that such capability will enable the design of truly scalable intelligent systems using neuromorphic hardware that embrace complexity in network dynamics rather than avoiding it.

## 1. Introduction

What role does the brain serve for producing adaptive behavior? This intriguing question is a long-standing one. So far, most attempts to understand brain function for adaptive behavior have primarily described it as the computation of behavioral responses from internal representations of stimuli and stored representations of past experience, a description we will take issue with below.

As computational systems have grown in functional complexity, the analogy between computers and the brain began to be widely adopted. The basic premise for this analogy was that both computers and the brain received information and acted upon it in complex ways to produce an output. This analogy between computers and the brain (also known as the computer metaphor) has provided a candidate mechanism for cognition, equating it with a digital computer program that can manipulate internal representation according to a set of rules.

The extensive use of the computer metaphor has resulted in the applied notions of symbolic computations and serial processing to construct human-like adaptive behaviors. The task of brain science has become focused on answering the question of how the brain computes (Piccinini and Shagrir, 2014). The key issues, such as serial vs. parallel processing, analog vs. digital coding, and symbolic vs. non-symbolic representations, are being addressed using the computer metaphor wherein perception, action, and cognition are taken to be input, output, and computation. Traditional algorithms that are derived by adopting the computer metaphor have yielded very limited utility in complex, real-world environments, despite several decades of research to develop machines that exhibit adaptive behaviors.

This impasse has forced us to rethink the notion of how adaptive behavior might be realized in machines. One inspiration comes from a key observation that was made in 1950s by Ashby (1947), when he designed a machine called the homeostat (Ashby, 1960). According to him, animals are driven by survival as the objective function and animals that survive are very successful in keeping their essential variables within physiological limits. The term homeostasis dates from 1926, when Cannon (1929) used it to describe the specialized mechanisms unique to living systems which preserve internal equilibrium in the case of an inconstant world (Moore-Ede, 1986). These variables and their limits are fixed through evolution. For example, in humans, if the systolic blood-pressure (which is an example of an essential variable) drops from 120 mm of mercury to 30, the change will result in death. Ashby's thesis then was that systems that exhibit adaptive behaviors are striving to keep their essential variables within limits. Our take-away was that machines that exhibit adaptive behaviors are like a control system that strives to keep a variable within or around a set point.

A related observation is that the complexity of adaptive behaviors increases with the number of physiological parameters that are to be maintained within their limits. We believe that this includes collective essential variables that are learned during the animal's interaction with the environment. However, approaching this from a control system point of view, it can be interpreted as the system striving to maintain stability across a complex set of interacting or coupled control loops with several set points. It is known that maintaining stability of such complex networks with multiple set points is a non-trivial task (Buldyrev et al., 2010). A second non-triviality is due to the inherent delays associated with homeostatic mechanisms. These delays require that homeostatic processes are also predictive. Indeed, analysis of periodic variations in essential variables, such as plasma cortisol levels in humans, shows that the so-called responses of homeostatic mechanisms are largely anticipatory (Moore-Ede, 1986).

The homeostat can be thought of as a simple kind of self-organization. Once its structure is set, external forces interact with various internal forces that automatically balance and create feedback that affects those same external forces. A special kind of dynamic balance, one that persists through and because of constant change, is known as criticality (for the notion of criticality intended, see Bak et al., 1987; Beggs and Plenz, 2003; Legenstein and Maass, 2007). A critical system is poised to react quickly to deviations or perturbations, because of a different balance at the system level—between decay and explosion. Homeostatic balance is a balance of forces, while critical balance is a balance of the dynamics themselves. A system that contains both kinds of balance is typical of so-called self-organized criticality (SOC). A slight reorganization of SOC puts criticality under the control of a homeostatic balance. Such a system would be driven toward criticality.

## 2. Criticality as a set-point

The mammalian cortex is a complex physical dynamical system. Several lines of research (Linkenkaer-Hansen et al., 2001; Beggs and Plenz, 2003; Petermann et al., 2009; Shew et al., 2011; Tagliazucchi et al., 2012; Yang et al., 2012), demonstrate that spontaneous cortical activity has structure, manifested as cascades of activity termed neuronal avalanches. Theory predicts that networks at criticality or edge-of-chaos, maximize information capacity and transmission (Shew et al., 2011), number of metastable states (Haldeman and Beggs, 2005), and optimized dynamic range (Kinouchi and Copelli, 2006). The ubiquity of scale invariance in nature combined with its advantages for complex dynamics suggests that each of the foregoing properties would be beneficial for both models and artificial systems (Avizienis et al., 2012; Srinivasa and Cruz-Albrecht, 2012).

Evidence for such rich and ceaseless dynamics has been reported across spatial and temporal scales and contain both spatial and temporal structure (Beggs and Plenz, 2003; Kitzbichler et al., 2009; Shew et al., 2011; Tagliazucchi et al., 2012; Yang et al., 2012; Haimovici et al., 2013). Brain activity exists in a highly flexible state, simultaneously maximizing both integration and segregation of information, with optimized information transfer and processing capabilities (Friston et al., 1997; Bressler and Kelso, 2001; Shew and Plenz, 2013; Tognoli and Kelso, 2014). One remarkable feature of spontaneous neural dynamics is that they are present in all but the most extreme brain states (e.g., deep anesthesia, Scott et al., 2014) and have been observed across many different brain configurations (e.g., with different ages, structural differences across individuals and across species).

A range of theoretical models has aimed to simulate brain activity at a macroscopic scale and explain how dynamics occur (Deco et al., 2009; Cabral et al., 2011; Hellyer et al., 2014; Messé et al., 2014). They developed several measures to describe the rich dynamics observed, e.g., metastability (Friston et al., 1997; Bressler and Kelso, 2001; Shanahan, 2010) and the notion of criticality (Shew and Plenz, 2013). These models demonstrate the primary importance of the underlying structural network organization but other factors are also important such as neural noise, time delays, the strength of connectivity as well as the balance of local excitation and inhibition (Deco et al., 2014). While simulating these macroscopic networks, the desired characteristics of the network (i.e., rich and stable dynamics observed with fMRI data) only occur within a narrow window of parameters. Outside this window, models will often fall into a pathological state: either (i) no dynamics (global activity at ceiling or floor); or (ii) random activity with little or no temporal or spatial structure. This is in contrast to actual brain activity, which maintains non-trivial dynamics in the face of a changing external environment as well as structural changes (e.g., the configuration of the brain's structural connectivity or neurotransmitter levels during aging) and across development and different species. A theoretical account of how spontaneous dynamics emerge in the brain must therefore include mechanisms that regulate those dynamics.

One potential mechanism for maintaining neural dynamics is inhibitory plasticity. Recent computational work suggests that, at the neural level, inhibitory plasticity can serve as a homeostatic mechanism by regulating the balance between excitatory and inhibitory (E/I) activity (Vogels et al., 2011). Moreover, this form of inhibitory plasticity has been shown to induce critical dynamics in a mean-field model of coupled excitatory and inhibitory neurons (Magnasco et al., 2009; Cowan et al., 2013), exhibit robust self-organization (Srinivasa and Jiang, 2013), pattern discrimination (Srinivasa and Cho, 2014), and cell assembly formation (Litwin-Kumar and Doiron, 2014). These theoretical results suggest that inhibitory homeostatic plasticity may provide a mechanism to stabilize brain dynamics at the macroscopic level, and may be relevant for understanding macroscopic patterns of brain activity.

Given the benefits, it would be useful for networks to be able to maintain a state of criticality. What follows is a description of an artificial neural system that appears to do so through a process of self-organization. We modeled a recurrent EI neuronal network consisting of 10,000 leaky integrate-and-fire (LIF) neurons (Vogels et al., 2005) composed of 8000 excitatory (E) neurons and 2000 inhibitory (I) neurons with a fixed connection probability of 1%. Several physiological phenomena were also modeled: two types of synaptic current dynamics, excitatory (AMPA) and inhibitory (GABA); Short-term plasticity (STP) (Markram and Tsodyks, 1996; Tsodyks et al., 1998), affecting instantaneous synaptic efficacy; and spike-timing dependent plasticity (STDP) (Markram et al., 1997), affecting long-term synaptic strength such that synapses are strengthened when presynaptic spikes precede postsynaptic spikes, and weakened otherwise. There is abundant neurophysiological evidence for the role of STP and STDP in shaping network structure and dynamics (Markram et al., 1997; Bi and Poo, 1998; Young et al., 2007) For details about this network design, see Stepp et al. (2015).

**Figure 1:**
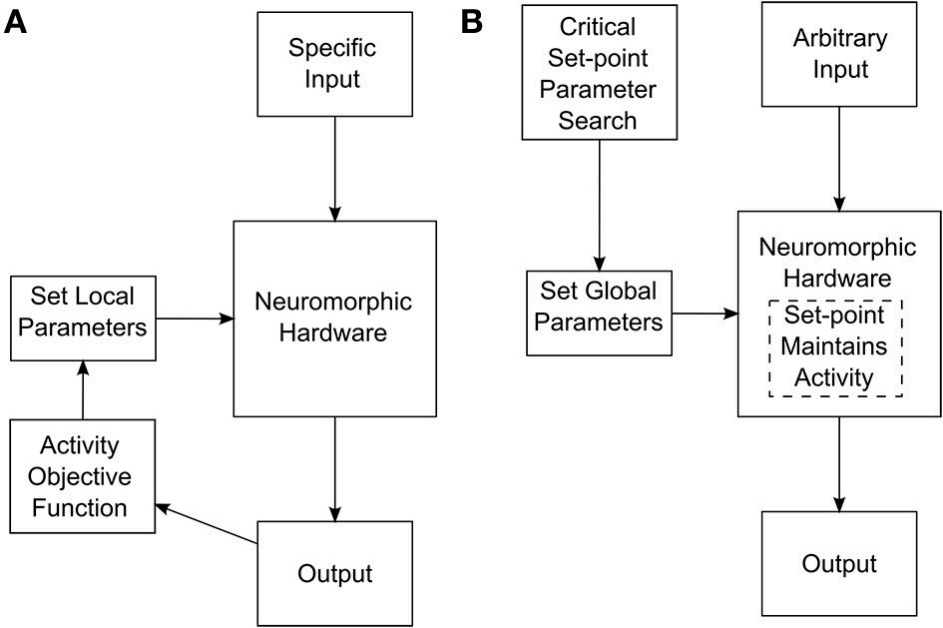
**(A)** Tuning process required if synaptic weights or other low-level parameters need to be tuned for specific network inputs. **(B)** Tuning process for a self-tuning critical network. In this case, the network is tuned at a high level to select for critical dynamics that is adaptively maintained internally.

To achieve a system that self-tunes toward criticality, i.e., treats criticality as set-point, we performed a parameter search at the level of global STP, STDP, and synaptic kinetics parameters. This search resulted in several network configurations that maintained themselves in a state of criticality, even after being perturbed by external inputs (For details about the parameter search and associated measurements, see Stepp et al., 2015). A parameter search of this sort should be distinguished from a search at the level of individual synaptic characteristics. For instance, tuning a network to maintain useful activity while receiving certain input might involve setting individual synaptic weights. These weights would generally have to be set differently for different classes of input. The search for self-tuning criticality happens at a more global network level, and the resulting network configuration is appropriate for many classes of input. The two different approaches are depicted in Figure 1.

For a system that must support adaptive behavior, criticality is directly beneficial. Critical systems, by virtue of their balancing act, have quick access to a large number of metastable states (Haldeman and Beggs, 2005). Being able to quickly diverge from one dynamical trajectory to another is clearly appropriate for a system that must adapt to a changing environment. Accordingly, the ability of non-autonomous neural circuits (i.e., ones that receive external inputs) to discriminate patterns tends to be increased for systems near edge-of-chaos states (Legenstein and Maass, 2007). In a recent work by our group (Srinivasa and Cho, 2014), we described a spiking neural model that can learn to discriminate patterns in an unsupervised manner. This can be construed as an adaptive behavior. For this network to exhibit proper function, we must have inhibitory plasticity, because it enables a dynamic balance of network currents between excitation and inhibition. It is during the transient imbalances in the currents that the network adapts its synaptic weights via STDP and thus learns patterns. The ability of the network to perform pattern discrimination (adaptive behavior) is impaired when inhibitory synaptic plasticity is shut off. A recent further study of this work (unpublished work) shows that this network exhibits avalanche dynamics when its spiking activity is evaluated based on the methods described in Stepp et al. (2015). Avalanche dynamics along with balanced inhibitory and excitatory currents strongly suggest that this network is critical. If implemented in neuromorphic hardware, it is then expected that it would function well in a configuration that seeks criticality, without needing an application-specific configuration. Adaptive behavior, to be truly adaptive, must also have some element of permanence. There is evidence that criticality could be a crucial ingredient for learning (de arcangelis and Herrmann, 2010). As such, the same feature appears to support both quick and lasting change, apropos for a phenomenon marked by a balance between opposing tendencies.

## 3. Neuromorphic hardware

There has been a recent interest in developing large scale neuromorphic electronics systems (DARPA SyNAPSE: http://en.wikipedia.org/wiki/SyNAPSE; Human Brain Project: http://www.humanbrainproject.eu/neuromorphic-computing-platform) to enable a new generation of computing platforms with energy efficiencies that compare to biological systems while being capable of learning from its interaction with its environment. For example, at the HRL Laboratories LLC, the multidisciplinary project funded by DARPA SyNAPSE (Srinivasa and Cruz-Albrecht, 2012) attempts to develop a theoretical foundation inspired by neuroscience to engineer electronic systems that exhibit intelligence. The ultimate goal of the project is to build neuromorphic electronics at large scales (for example with 10^8^ neurons and 10^11^ synapses) to realize autonomous systems that exhibit high combinatorial complexity in adapting to a large variety of environments.

A major problem recognized by the field in developing such large scale systems is the need for monitoring its dynamics for debugging purposes as well as to be able to tune the parameters of such a massive chip, which is currently not feasible due to inherent limitations in accessibility to all the chip components as well as the lack of a clear understanding how to design such self-tuning complex systems. In integrated circuit implementations of large neural networks it is useful, or even necessary, not to have the requirement to monitor all the internal signals of the network. The internal signals could include, for example, spiking signals produced by internal neurons (Cruz-Albrecht et al., 2013), and digital or analog weights of the synapses located between neurons (Cruz-Albrecht et al., 2012, 2013). To monitor the internal signals of the chip requires the use of circuitry connecting internal neurons and synapses to the terminals, or pads, of the chip. The monitoring circuits are detrimental to the density of the network, reducing the number of neurons and synapses that can be implemented for a given area of the chip. It is also detrimental to the efficiency of the network, reducing the number of neural operations per unit of time that can be performed by a given amount of electrical power consumed by the chip. As CMOS technology scales to smaller features, the number of neurons and synapses that can be implemented in a chip grows faster than the number of chip terminals. Similarly the internal bandwidth (Zorian, 1999) of the chips is expected to increase faster than the interface bandwidth (Scholze et al., 2011) with scaling. Therefore, as technology scales it is expected that a smaller portion of all the internal signals of large chips can be monitored simultaneously in real time. If the neural chip is more analog in nature (Cruz-Albrecht et al., 2012), these problems described above are further exacerbated.

**Figure 2:**
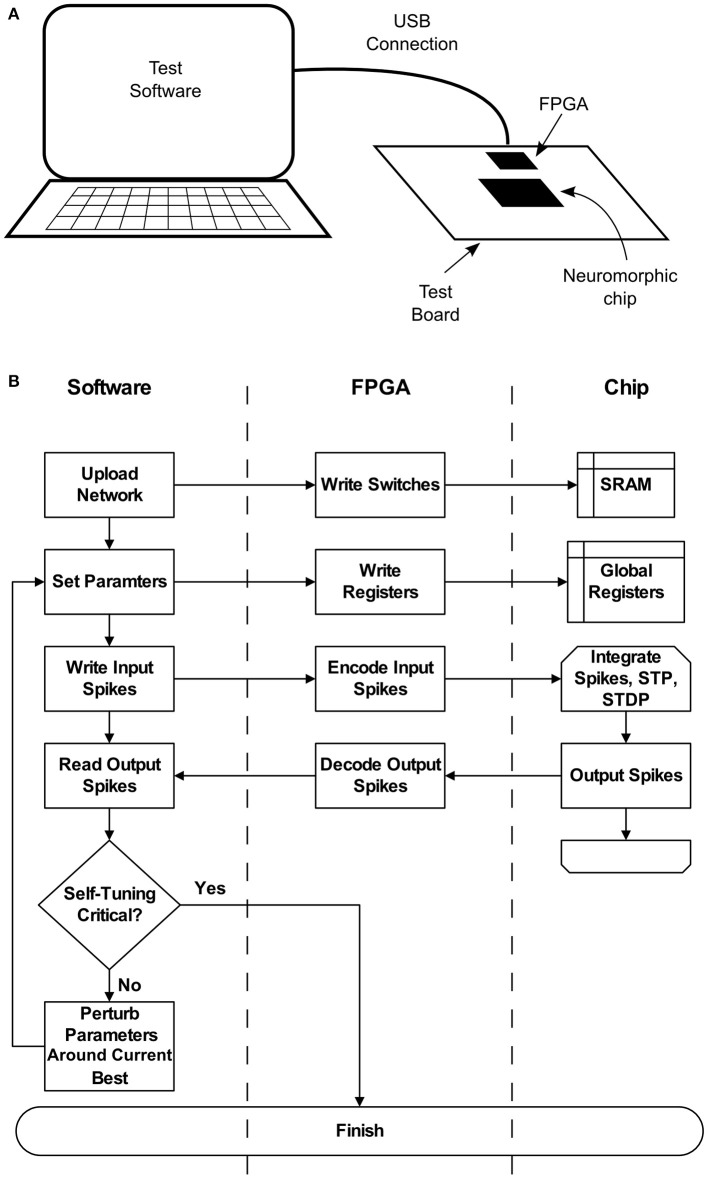
**(A)** A typical configuration for interacting with the neuromorphic hardware, for instance when conducting a parameter search. Test software runs on a general purpose computer, which communicates with an FPGA over a USB connection. The connection allows software to upload networks to the chip, set hardware parameters, and perform spike-based input and output. **(B)** Flow chart detailing the parameter search process and its relation to each system component.

These deficiencies may be addressed if the chip can be set up to operate with criticality as a set-point. To achieve this, a high-level parameter search similar to the one described in Stepp et al. (2015) can be run on the hardware itself. Figure 2 depicts a typical setup, where a neuromorphic chip is installed on a test board, along with a supporting FPGA. A general purpose computer communicates with the FPGA via a USB connection, which enables software to configure the chip as well as send and receive spikes. Once a network is uploaded, parameters can be quickly set and re-set by modifying on-chip registers. A typical search requires less than 100 iterations of 300 s each, amounting to a worst-case runtime of approximately 8 h. This search would result in a configuration that could be set once, without requiring access to low-level parameters such as synaptic weights. Self-tuning criticality, again as shown in Stepp et al. (2015), would then ensure that the network maintained a useful level of activity without input-specific tuning. If parts of the hardware break or begin to function differently, we expect an amount of fault tolerance. Without respect to self-tuning criticality, neural networks of this sort are already relatively tolerant (Srinivasa and Cho, 2012). Beyond this intrinsic fault tolerance, the self-tuning aspect described here extends this capability. At some point, however, the network dynamics will become too different and the search will have to be repeated. The nature of this breaking point is not well understood, and is a subject for further study.

We believe that this will result in a novel paradigm for computing and enable the design of a wide range of systems from small to large scales that exhibit robust adaptive behavior in the face of uncertainties. This may also lead to large scale neuromorphic system designs that more accurately account for brain-like dynamics compared to current designs (Eliasmith et al., 2012). Finally, it could also enable the design of truly scalable intelligent systems, since there will not be a need for manual tuning of model or chip parameters by constructing self-tuning critical networks that in turn will enable adaptive behaviors.

## Author contributions

NS conceived of the work and drafted the manuscript, NDS drafted the manuscript and performed analyses, JC drafted the manuscript.

## Conflict of interest statement

The authors declare that the research was conducted in the absence of any commercial or financial relationships that could be construed as a potential conflict of interest.
